# The Intentional Selection Assumption

**DOI:** 10.3389/fpsyg.2021.569275

**Published:** 2021-10-26

**Authors:** Joseph Colantonio, Kelley Durkin, Leyla Roksan Caglar, Patrick Shafto, Elizabeth Bonawitz

**Affiliations:** ^1^Department of Psychology, Rutgers University—Newark, Newark, NJ, United States; ^2^Peabody College of Education and Human Development, Vanderbilt University, Nashville, TN, United States; ^3^Department of Mathematics and Computer Science, Rutgers University—Newark, Newark, NJ, United States; ^4^Graduate School of Education, Harvard University, Cambridge, MA, United States

**Keywords:** decision making, social cognition, social, intentional selection, context

## Abstract

There exists a rich literature describing how social context influences decision making. Here, we propose a novel framing of social influences, the Intentional Selection Assumption. This framework proposes that, when a person is presented with a set of options by another social agent, people may treat the set of options as intentionally selected, reflecting the chooser's inferences about the presenter and the presenter's goals. To describe our proposal, we draw analogies to the cognition literature on sampling inferences within concept learning. This is done to highlight how the Intentional Selection Assumption accounts for both normative (e.g., comparing perceived utilities) and subjective (e.g., consideration of context relevance) principles in decision making, while also highlighting how analogous findings in the concept learning literature can aid in bridging these principles by drawing attention to the importance of potential sampling assumptions within decision making paradigms. We present the two behavioral experiments that provide support to this proposal and find that social-contextual cues influence choice behavior with respect to the induction of sampling assumptions. We then discuss a theoretical framework of the Intentional Selection Assumption alongside the possibility of its potential relationships to contemporary models of choice. Overall, our results emphasize the flexibility of decision makers with respect to social-contextual factors without sacrificing systematicity regarding the preference for specific options with a higher value or utility.

## Introduction

Many factors can influence our daily decisions. These factors may include the many ways that potential choices are presented to us, how many options are among those potential choices, and the possible features on which these choices vary. So, how might people choose one particular option when presented with many? For example, when only presented with two options for a news subscription such as a physical, printed copy, or a digital, online version, which option should you choose? Here, people may act systematically—considering the comparable features of the objects presented (such as the cost of each subscription, the desire for physical accessibility vs. online, etc.). However, people may also consider the context of this decision-making scenario. For example, they may make inferences about the process by which these options became available such as whether another person intentionally chooses these two subscription options in relation to their thoughts or goals. Together, the interaction between the formerly mentioned comparison of fixed feature values and the latter moderation based on the subjective influences that typically arise from social situations may make it difficult to decide what may be the best option to choose.

This overarching question of how people make choices is not new and has been explored in many disciplines—including psychology, philosophy, economics, marketing, and computer science. Such interest has led to a variety of theoretical and formal models of choice behavior dating back more than 100 years (e.g., Thurstone, 1927; Luce, 1959; McFadden, 1977; Yellot, 1977; Sutton and Barto, 1998). Thus, decision-making theories and models have many tenets—sometimes contrasting with one another on what aspects of decision-making scenarios matter the most. For example, some context-free models of choice rely strictly on normative principles (Luce, 1959; for example, see Echenique and Saito, 2018; Kovach and Tserenjigmid, 2018), whereas other models consider close attention to contextual or subjective influences (Busemeyer and Rieskamp, 2014; for example, see Mosteller and Nogee, 1951; Hey, 2001; Feng et al., 2018). For example, Luce (1959) proposed the Luce choice rule, a foundation for normative principles of decision-making, which posits that choice behavior is systematic and probabilistic. Currently, the Luce choice rule suggests that the probability of choosing an option is proportional to its perceived utility, relative only to the other presently available items (and no other items) and that these relative proportions are held across all sets of items regardless of context. This normative approach is favorable at times because it provides a straightforward implementation of the models that are still influential today (Busemeyer and Rieskamp, 2014).

However, while decision-making does incorporate the use of probabilistic information, empirical work has demonstrated for decades that humans exhibit contextually flexible choice behavior (e.g., Tversky, 1972; Simonson and Tversky, 1992). In fact, some proponents of subjective accounts had proposed that people do not always choose in proportion to probabilities (e.g., Debreu, 1960; Tversky, 1972; Simonson and Tversky, 1992), nor do people reflect any stable concept of utility or weights across contexts (Rieskamp et al., 2006). The notion that people are sensitive to context can make the design and investigation of formal frameworks, theories, and models of choice behavior difficult. Empirical accounts may point out behavioral nuances that draw from both normative and subjective principles as some systematicity exists in decision-making. We view this problem as an explanatory opportunity for an understanding of the nuances of decision-making across varied contexts. That is, how might we understand the systematic variability of choices (given specific contexts) while still accounting for tendencies toward probabilistic reasoning presented in recent decision-making research? Here, we focus on one particular component of this process: social context.

This notion of social-contextual choices is grounded in a few previous literature studies but may require additional bridging between the two closely related subfields of cognition and psychology—decision-making and concept learning. Firstly, a previous work in decision-making has proposed that social-contextual cues may serve as an additional important factor in decision-making (Thaler and Sunstein, 2008; Moshinsky and Bar-Hillel, 2010; Johnson et al., 2012). We extend this work by investigating a specific aspect of decision-making scenarios that are less understood, suggesting that people may also consider whether the available options in a decision-making scenario were *intentionally* sampled for them by a social agent. This proposal extends the rich past work and a growing interest of context-dependent research by specifically considering the generative process of the available choice options: when assuming the intentional sampling of options provided by a social agent as the presenter, a decision maker may then make inferences about the goals of the presented options and about why those samples are presented (as opposed to the inferred alternatives not presented).

Thus, to support our notion of the importance of investigating option sampling and generation, we first describe research on decision-making—in particular, a work that highlights “anomalies” of rational choice per strictly normative principles. Then, we discuss how the key findings in the concept learning literature may lend themselves in explaining the less understood aspects of decision-making—the generation and presentation (read as “sampling”) of options that a decision maker may choose from. Finally, we detail the two experiments that provide empirical evidence in support of our proposed theory within decision-making: the Intentional Selection Assumption.

### Choice in Context

Decision-making theories face the challenge of generalizing the choice formally while still addressing the importance of context. This challenge itself is not new as early empirical evidence suggests that human choice behavior is not independent of alternative options (Tversky, 1972; Simonson and Tversky, 1992; Tversky and Simonson, 1993; Ariely, 2000). Furthermore, more recent research also highlights the idea that “rational decisions” are not limited to the strict definitions of choice as implied in traditional normative views of decision-making theories (see Rieskamp et al., 2006; Busemeyer and Rieskamp, 2014 for a further overview). For example, Huber et al. (1982) report on an asymmetric dominance effect, often called the Attraction or Decoy effect, a classic example cited in the decision-making literature. Here, participants have to choose between items (e.g., the brands of beer) that vary along two dimensions (e.g., price and quality of taste). In a no-decoy condition, participants have to choose between the two items that vary “equally” by each feature (e.g., one beer that costs more but tastes better vs. another that costs less but has a lower quality). In a decoy condition, a third option that does not improve on one dimension and is more costly on another is added (e.g., a beer of similar quality to a better quality option but costs substantially more). When this decoy option (that no one chooses) is added, it increases the “value” or choices toward the item that dominates on that feature.

It is becoming increasingly important to address and explain decision making anomalies as a result of contextual variability, as this effect of context on choice behavior illustrates that choice depends not only on normative weights of options, which are assumed to be independent of each other. This is because when asking an individual to decide, the methods for presenting information about available options may be perceived or inferred by a decision maker (Johnson et al., 2012). Research on the effects of the elements of choice architecture on behaviors has grown in recent decades; investigation of the nuances of decision paradigm designs that influence decisions has been conducted. This includes aspects of the paradigm such as the number of available alternatives (Cronqvist and Thaler, 2004), the presence of defaults (Johnson and Goldstein, 2003), the categories in which the options are grouped (Fox et al., 2005), and the units used to describe attributes (Larrick and Soll, 2008). Thus, in describing our theory of the Intentional Selection Assumption, we also need to acknowledge other informational theories and accounts of decision-making as they look into explaining how these variations in choice presentation (and thus choice context) combine classical normative expectations with subject contextual influences.

Following from the “options-as-information” theory (e.g., Sher and McKenzie, 2006; Müller-Trede et al., 2015), challenges to the classical decision theory arise from violations to strictly normative expectations found in a few literature studies when participants make choices across different contexts. According to this and other informational accounts, there may be additional information inferred by the participant across the decision-making scenarios that vary in context despite having equivalent information among the literal contents of the options presented. Thus, this “information leakage” (as Sher and McKenzie, 2006 refer to it) is problematic for rational models of decision-making. Furthermore, a previous work on the effects of option salience by Wernerfelt (1995) alongside a more recent analysis by Kamenica (2008) and Bordalo et al. (2013) offers further insights into the benefits of an informational account that investigates feature and option salience. Specifically, this work looks into describing how variability in the salience of available options to choose from may also affect the amount of information given to a decision maker, such as in the Decoy effect (Wernerfelt, 1995). Thus, when salience is manipulated (intentionally or not) and varied information is provided across scenarios, decision makers may follow suit and vary their own inferences of the values and relevance of the available options. This, of course, is based on whether they also infer that the provided “decoy” and variance in option salience are intentional and thus making them relevant.

Here, we propose to investigate a previously unexplored element of choice: the assumptions of decision makers regarding option sampling and generation. Both foundational research on choice and recent empirical investigations have made either explicit (mathematical) assumptions about sampling (as far back as Luce, 1959) or lacked the descriptions regarding the sampling, generation, and presentation of options. Specifically, past research houses implicit the assumptions that random sampling has occurred and is similarly inferred by both the presenter (the experimenter) and the decision makers (the participants). This assumption of random sampling implies that current options are independent of nonpresented options and that subsequent options are generated without knowledge or dependence on the options that are already drawn, lending itself to “rational” decision-making relying only on normative principles. However, as shown by the myriad of a recent experimental work that highlights a variation from normative expectations, the manipulations of the experimental paradigm may also affect participants' inferences that are made about the sampling process. Thus, we propose to bridge a few rich literature studies on concept learning—where sampling assumptions are regularly disclosed and carefully moderated—to investigate this variation of choice behavior as described in the decision-making literature.

### Intentional Selection and Sampling: Analogous Findings From Concept Learning

As mentioned earlier, computational models and empirical results in the concept learning literature suggest that participants do not always assume normative properties such as random sampling (Tenenbaum and Griffiths, 2001; Xu and Tenenbaum, 2007a,b). Instead, it has been argued that individuals bring different assumptions to bear on data that are generated by another person for a specific reason, such as “strong sampling” or teaching a new concept (Tenenbaum and Griffiths, 2001; Shafto and Goodman, 2008; Shafto et al., 2014; Bonawitz and Shafto, 2016).

Some studies on concept learning demonstrate that people can make inferences about Strong Sampling—showing sensitivity to whether or not examples are generated purposefully within a target concept (Tenenbaum and Griffiths, 2001; Xu and Tenenbaum, 2007a,b). For example, Xu and Tenenbaum (2007a) manipulate whether the examples in a word learning task were given by a knowledgeable or naive generator. Critically, the examples were matched between conditions. However, because knowledgeable samplers are choosing the examples from within the concept, the principles of strong sampling apply. In contrast, naive samplers are necessarily generating samples from the full hypothesis space—consistent with weak sampling. Learners in Xu and Tenenbaum (2007a) had a tendency to generalize differently from these events. This suggests that, in different learning contexts, learners consider the process by which the examples are generated and adjust inferences accordingly.

Another concept learning research has conceptualized an additional kind of intentional sampling, pedagogical sampling, where a “teacher” provides the samples that maximize the chances of the learner inferring the correct concept. Critically, in this model, the learner reciprocally assumed that the provided samples were pedagogical and thus maximally “informative” of the target concept. The consequence of this joint inference between a teacher and learner is a rapid constraint of hypotheses as observed in the studies of adults (Tenenbaum and Griffiths, 2001; Shafto and Goodman, 2008; Shafto et al., 2014) and children (Bonawitz et al., 2011, 2020) learners.

### Sampling Assumptions Within Models of Choice Behavior

While research in the concept learning literature has demonstrated the relevance of sampling assumptions in the studies of human inference and learning (Shafto et al., 2012), work on decision-making does not fully account for the influences of these powerful sampling assumptions. Meanwhile, decision-making research has a history of investigating the effects of context (e.g., recently within the Similarity Effect: Soltani et al., 2012), and some models of choice do show an ability to predict the influence of context, framing, or pragmatics, they tend to implement bias toward (or inhibition against) past preferences (e.g., Roe et al., 2001; Usher and McClelland, 2004; Bhatia, 2013) with strong sampling assumptions (Tenenbaum and Griffiths, 2001; Xu and Tenenbaum, 2007a,b). For example, Shenoy and Yu (2013) present a model of the aforementioned effects, with assumptions similar to our theory of Intentional Selection. Specifically, they highlight that feature relevance is based on an individual's perceived “fair market value.” However, this model still relies on the strong assumptions of the choice generating process, such as the previously mentioned prior bias and random sampling.

More recent work provides some empirical evidence and computational accounts of canonical effects in the decision-making and learning literature which are sensitive to sampling assumptions. For example, Hayes et al. (2019) highlight the importance of sampling processes (strong vs. weak) by outlining both a Bayesian model and the empirical investigation of the diversity effect (see Osherson et al., 1990). Similarly, Lee et al. (2019) investigate the diversity effect and present evidence on the importance of sample “closeness” or a variance in an associative learning task, however, without a focus on the sampling process.

Here, we propose that human behavior as described in the decision-making literature (e.g., such as the Attraction Effect) may be affected by the Intentional Selection Assumption—a novel factor that integrates social cognitive cues into decision-making. According to the Intentional Selection Assumption, the inferred goals and beliefs of the provider impact the perceived utility of features among the selected options. Borrowing from subjective accounts, the relative utility afforded to options may vary across changes in social context as affected by the Intentional Selection Assumption. However, principles from normative accounts—such as choosing the perceived best option within said social context—should still affect decision-making among intentionally selected options. Thus, our approach includes a stable notion of utility that could vary with changes in a social context, affording a means to capture the aspects of both normative principles and subjective, contextual influences.

Critically, this Intentional Selection Assumption entails the description of how decision makers make inferences about how the presenter generates the set of available options for a decision maker to choose from—an aspect of the choice scenarios that may often be overlooked. For example, many past behavioral experiments attempt to utilize random sampling when presenting choice tasks to participants to prevent bias, assuming that participants would also assume random sampling. Thus, they may not mention the process by which the options are selected or explicitly assume that the options presented are randomly sampled. Indeed, many models directly or indirectly assume the independence of irrelevant alternatives (Arrow, 1963). This raises the question of how people actually weigh the available options without restricting themselves to fixed normative or strictly context-specific values.

From a work on concept learning discussed earlier, we see evidence that people do not display the behavior that reflects the assumptions of random sampling as a default. Thus, one possibility is that we can understand behavior in previous decision-making tasks as similarly reflecting sampling assumptions inconsistent with random sampling. That is, in the absence of information about how or why options are being presented in an experimental paradigm, participants may not default to random sampling assumptions. Instead, the context of the experiment itself may lead participants to infer a process of intentional sampling for the presented options.

Indeed, since the establishment of foundational work on decision-making (e.g., Luce, 1959), several lines of research have pointed either indirectly or directly to the role of social context and Intentional Selection in driving choice behavior. One example of how social context influences decision-making is found in the modified versions of the Ultimatum Game (FeldmanHall et al., 2014). Here, participants are told they are responsible for determining another person's punishment for committing a selfish action. In past versions, participants chose a form of punishment from a pair of options: punish the selfish agent or do nothing. This set of options led to significantly more decisions to punish than do nothing. In a modified version of this task, FeldmanHall et al. (2014) included additional options, such as imposing equity or compensating the victim of the antisocial behavior. They found that participants with more available options preferred restorative as opposed to punitive options, suggesting a change in the context of the task. The implied “ultimatum” may have been imposed by the experimenters using the original version and not by the participants who have made the decisions. These results are consistent with the idea that having choices provided for you can influence a preference. However, this research did not set out to test the Intentional Selection Assumption and so would not control for obvious alternative interpretations (that the better options were not obvious to participants and simply providing them should thus lead to their choice).

Another work related to the Intentional Selection Assumption shows how the social cue of pragmatics plays a role in models of choice. Specifically, how pragmatics can change how individuals frame choice scenarios, appraising, or attending to specific information as relevant based on their own inferences (e.g., Hilton, 2002, 2017), and much recent work describes how pragmatics leads to framing effects that explain “irrational” choices regularly seen in empirical studies (e.g., Sher and McKenzie, 2006; McKenzie et al., 2018). Furthermore, some recent works by Leong et al. (2017) find that despite the equivalence of the presented information in two scenarios (e.g., hypothetical basketball players that either make 40% of their shots or miss 60% of them), these differently framed instances of equivalent information elicit different appraisals of the same player's relative skill level (above average or below average, respectively). These pragmatic and social influences are similarly found in a work on “nudge theory,” which proposes that positive reinforcement and indirect suggestions can be used to influence behavior and decision-making (Tannenbaum et al., 2014, 2017). Similarly, Bless and Schwarz (2010) present the Inclusion/Exclusion model - which considers the importance of both pragmatics and social, conversational cues in information transmission.

Furthermore, some recent research studies have looked into addressing the roles that an option presenter may have in influencing the perceived values and final decisions of choosers. For example, Basu and Savani (2017, 2019) acknowledge the importance of framing effects specifically in regard to option presentation. Specifically, the authors find that whether the options are presented one-by-one or all-at-once (simultaneously) affects how “optimally” participants make decisions such that simultaneous presentation of all available options encourages more optimal decision-making with these participants more often choosing a “dominating” option (per normative standards), compared to participants viewing options sequentially. However, in this work, there still lacks the distinction of how these samples are generated: as mentioned when discussing a prior work there may be the assumptions of random selection or sampling.

Thus, while the work discussed now is not exhaustive of the vast decision-making literature, it is clear that much work has been completed in disentangling several different social-cognitive processes that support human inference (see also Waytz and Mitchell, 2011). Furthermore, these works provide insights into the importance of context and social cues and have been integrated into formal models of decision-making by importantly considering how inferences (e.g., framing) lead participants toward one choice over another. However, while this past work contributes largely to the importance of addressing “irrational” human choice, it does not yet investigate how the purposeful generation of the available choices affects decision makers' inferences during this process. Here, we argue that investigating how decision makers' inferences in regard to the sampling of their presented choices could shed light on at least one specific way in which social context and the selection of options influence decision-making: through a purposeful selection of information. Specifically, we focus on investigating the aspects of how and why the options are presented by social (as opposed to non-social) agents as additional factors in extending past decision-making theories. Here, we demonstrate that people can infer that choice options were intentionally chosen for them by a social agent with a specific goal in mind and refer to this inference as the Intentional Selection Assumption (Shafto and Bonawitz, 2015).

### The Intentional Selection Proposal

We see our proposal—that choice behavior may depend on an “Intentional Selection Assumption”—as consistent with the past literature of social pragmatics and framing in choice behavior. We posit that, when people are presented with multiple options, they may assume that the options were selected intentionally by an agent with a specific goal. This sets forth the chooser's inferences about relevant features based on the contextual factors of the choice scenario. As the chooser considers the relevance of features, they may calculate relative utilities based on their inferences of the presenter's goals and beliefs—following normative principles to make decisions based on the perceived values. However, a chooser may still infer the value of each option and its features in respect to the choice scenario—allowing subjective, contextual effects to alter their decisions. From this, we see that we can draw on normative principles as we consider stability across features. However, we also lean on the theory from subjective accounts as there is no fixed utility for an option universally across scenarios, and there may be uncertainty about the relevance of features in a particular context.

Following the findings in the concept learning literature, when presenting individuals with the options from which they have to choose, a chooser may infer that the questioner's actions are goal-directed and will reason about why they do the things they do. This has implications for the types of inferences that the chooser can draw and how the chooser may assign values to various options and their features whether it involves the goals of the questioner, the context of the situation, or the potential utility of the options that are presented. As a result, the explicit assumptions of past normative accounts (Luce, 1959) face problems as they claim to be independent between observed and unobserved options in choice scenarios. Due to this normative assumption, there may have been no analysis for the effects of option relevance as shown by the lack of adherence to simple principles of decision-making (e.g., context-independent).

Our empirical contribution is a test of our theory of Intentional Selection in the choice domain, where we present two experiments centered on the effects of social context on this decision-making behavior. We take a novel approach to typical studies of choice behavior. Here, we manipulate whether the options are generated intentionally or accidentally (randomly) for participants. This provides critical evidence to determine whether social cues from the Intentional Selection of options are the factors in human choice behavior. We conclude with future directions related to decision-making as explored from a social-cognitive perspective.

## Empirical Evaluation

Thus, to support our proposed Intentional Selection Assumption, experiments were designed, which would highlight how choosers inferred that the individual (a social agent) selecting the available options did so purposefully and optimally, with a specific goal in mind. Furthermore, such assumptions from the chooser would not hold if the items were instead being chosen randomly (e.g., the options are not provided by a purposeful agent). Within the Appendix^1^, we provide a simplified demonstration of how the Intentional Selection Assumption explains differences in chooser's inferences about another person's beliefs.

Our primary studies examine how the Intentional Selection Assumption affects varying contexts and features (including a baseline control to replicate past findings in the decision-making literature; Ariely, 2000). Importantly, in our experiments, we include the conditions where the presented options are either chosen intentionally by another social agent (such as a friend or marketplace; the Intentional condition) or accidentally provided (the Accidental condition) and investigate whether participants are sensitive to these contextual differences in the option generation process—affecting their choice preferences. Thus, by comparing participants' behavior between conditions, we can investigate whether the decision makers are sensitive to sampling assumptions (as exhibited *via* the existence of social cues) as implied by the Intentional Selection Assumption.

### Materials and Methods: Experiment 1

**Figure 1:**
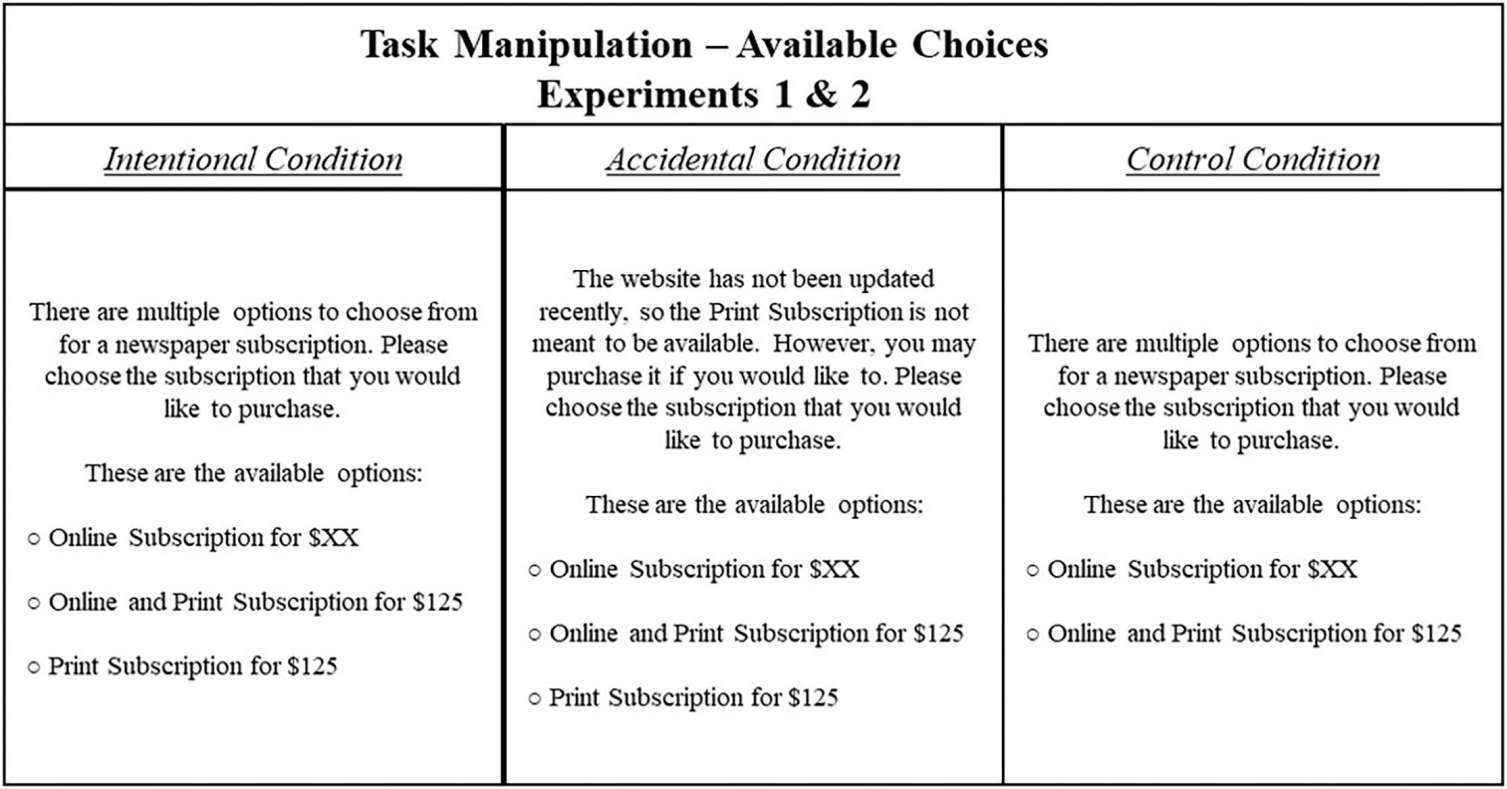
Task manipulation performed in Experiments 1 and 2. Here, participants completed an online survey similar to a past work on the relativity of available choices (see Ariely, 2010). In all conditions, participants are presented with multiple options for purchasing a newspaper subscription: access to an online-only version, access to a joint online-and-print version, and (within the Intentional and Random conditions, only) a print-only version. Importantly, the presentation of these options varied across conditions in regard to the print-only version—with a purposeful inclusion in the Intentional condition, an unintentional inclusion in the Accidental condition, and an exclusion from the Control condition. Between experiments, the price of the online-only subscription was changed. In Experiment 1, $XX = $59. In Experiment 2, $XX = 99.

The primary goal of Experiment 1 was to test our Intentional Selection model with a classic finding in the choice literature, the Attraction Effect. However, our design differs from past research on the Attraction Effect (e.g., Huber et al., 1982; Pettibone, 2012; Bhatia, 2013) as we vary the social context across each of our conditions to determine said context's effects. The visualization of both experiments can be found in Figure 1.

#### Participants

About 112 workers were recruited from Amazon Mechanical Turk. A payment of $0.50 was offered for the completion of this study. Six participants failed to pass an attention check and were therefore excluded from any analysis. The 106 remaining participants were randomly assigned to one of the three experimental conditions: the Intentional condition (*n* = 36), the Accidental condition (*n* = 35), and the Control condition (*n* = 35).

#### Methods

The task involves participants choosing one of the three different media of newspaper subscriptions that vary on price and the type of access (*via* print and/or online). We contrast the three conditions in which the availability of the options varies (Figure 1). Firstly, in the Intentional Condition, three subscription options are intentionally presented and available for purchase (an online subscription for $59, a print subscription for $125, and a joint online and print subscription for $125). In the Accidental condition, the print-only option is unintentionally presented but still available for participants to choose. Specifically, participants in the Accidental condition were informed that the website from which they were purchasing had accidentally not been updated and that the print-only option was not meant to be available for purchase (but could otherwise be purchased if they wanted).

Given that all options in the Intentional condition were intentionally presented to the participant, we predict that the pattern of subscription choices made by participants in the Intentional condition would be significantly different from the choice patterns made by the Accidental condition. Specifically, because the inclusion of the print-only option was unintentional in this condition, we predict that participants should infer that the price of this option is not actually representative of its relevance and utility, compared to participants in the intentional condition where the print-only option is intentionally presented.

We also include a baseline Control condition where participants were only presented with two of the abovementioned options: the online subscription for $59 or the joint online and print subscription for $125. This Control condition controls the inclusion of the print-only subscription in the other two conditions. Because the print-only condition is not at all available for these participants, they should infer that the value of print access is not as important or as relevant as it is in the Intentional condition. Additionally, because the participants in the Control condition are not given information on the value of print access, they should similarly perceive it as not relevant when compared to the Accidental condition. Therefore, we predict that choice behavior in this Control condition should significantly differ from the Intentional condition but not from the Accidental condition.

Importantly, the respective pricing options were chosen with respect to past research on the “Decoy” or “Attraction” Effect (Feng et al., 2018), where the presence of an intermediary (but asymmetrically dominated) option has been found to influence decision-making. By including the “decoy” of the print-only subscription, we can infer whether the difference in context between conditions affects participants' appraisals of the different features of the available options (access to print and access to online) *via* their final purchase decisions. The fact that the print-only option happens to be listed at the same price as the joint option is of little concern, as the primary manipulation is whether the print-only option was *intended* to be offered. If participants in the Intentional condition infer that the provider has the goal of intentionally including the print-only option so that potential subscribers may see (and perhaps purchase) it, they may attribute a higher value to print access as a feature and purchase options including it—even at a higher financial cost. This would be compared to the scenario in the Accidental condition where the presenter explicitly wanted to omit the possibility of a print-only option or compared to the Control condition, where a print-only option may not have even been considered at all. In these two latter contexts, inferences of the presenter's goals may lead to participants' inferences of print access as low in value or even irrelevant, leading to fewer decisions to purchase options with print access. Thus, it is important to keep in mind that a key difference between this current work and past studies on the Decoy Effect comes from the contrasts in option presentation between conditions as described earlier.

#### Results

**Figure 2:**
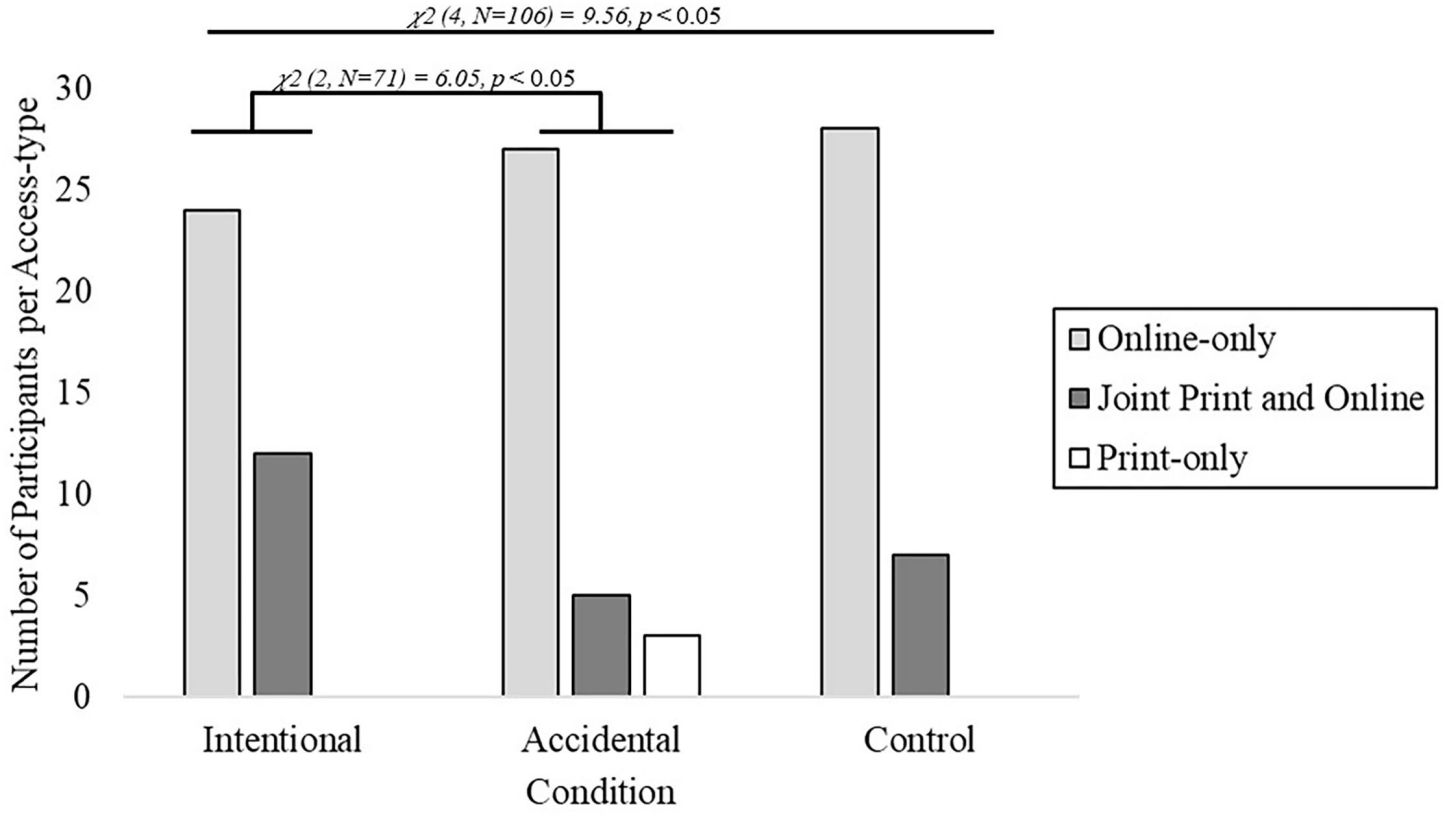
Forced choice results obtained from Experiment 1. Participants were more likely to choose the joint print and online subscription option in the Intentional condition compared to Accidental and Control conditions, χ^2^ (4, *n* = 106) = 9.56, *p* = 0.048.

The number of participants who chose each option differed significantly across the three conditions [the chi-squared test of proportion; χ^2^ (4, *n* = 106) = 9.56, *p* = 0.048], with participants in the Intentional Selection condition more likely to choose the joint online and print subscription (see Figure 2). Importantly, we find support for our first prediction—such that Intentional Selection and Accidental conditions were significantly different from one another [the chi-squared test of proportion; χ^2^ (2, *n* = 71) = 6.05, *p* = 0.049] despite both conditions having identical options available.

When further comparing the choices that are made of the two shared options (online-only and joint online and print subscriptions) across conditions, three additional chi-squared tests were performed. Here, we find that the Intentional condition has a marginally significant difference from the Accidental condition [the chi-squared test of proportion; χ^2^(1, *n* = 68) = 2.89 *p* = 0.07], and has no significant difference between Intentional and Control conditions [the chi-squared test of proportion; χ^2^ (1, *n* = 71) = 1.0, *p* = 0.32] nor between Accidental and Control conditions [the chi-squared test of proportion; χ^2^ (1, *n* = 37) = 0.02, *p* = 0.88]. Furthermore, after applying a Bonferroni correction for each of these pairwise comparisons (approximate α = 0.05/3 tests = 0.167), none of the comparisons are significantly different.

Investigating our second prediction, we separately compared the choices that are made by participants in the two experimental conditions (Intentional and Accidental) to the choices that are made by participants in the Control condition. Here, if participants infer that access to the newspaper through the print medium is relevant and useful in the Intentional condition, we predict that they should choose the joint subscription more often. However, if participants do not infer that print access is important, we predict they will forego the joint subscription and only purchase online access. Here, we further compared a preference between Intentional and Accidental conditions by determining the ratio of choices between online-only and the joint online and print subscriptions across the three conditions, and compared the two experimental conditions first against each other and then against the Control condition. Using Fisher's Exact test, we find a marginal difference between Intentional (choice ratio 27:5) and Accidental (choice ratio 24: 12) conditions (one-tailed, *p* = 0.07). However, neither the Intentional condition (one-tailed, *p* = 0.15) nor the Accidental condition (one-tailed, *p* = 0.44) was significantly different from the Control condition (choice ratio 28:7).

Importantly, Experiment 1 finds that there seems to be a difference (albeit marginally) specifically between Intentional Selection and Accidental conditions. This leaves open questions as to whether there may be factors within the decision-making scenario that may be moderating the strength of the Intentional Selection Assumption, such as feature variability (e.g., the variability of option pricing). Thus, to test the robustness of an effect, we conducted Experiment 2 to replicate and extend this work.

### Materials and Methods: Experiment 2

#### Participants

About 100 workers were recruited from Amazon Mechanical Turk. Three participants failed to pass an attention check and were therefore excluded from any analysis. The 97 remaining participants were randomly assigned to one of the three experimental conditions: the Intentional condition (*n* = 32), the Accidental condition (*n* = 32), and the Control condition (*n* = 33).

#### Methods

Experiment 2 aimed to provide further support that the Intentional Selection of the presented options, as contrasted with the accidental presentation of the same options, may have led to differences in decision-making among the conditions that could possibly be moderated by the pricing of said objects. Thus, the design of Experiment 2 was nearly identical to Experiment 1 (Figure 1) but with two key differences. Firstly, we raised the price of the online-only subscription from $59 to $99. This was changed as we believed that making the price feature of options more similar would increase the differences in choice behavior between Intentional and Accidental conditions. This is important to address as it may be an important aspect of our proposed Intentional Selection Assumption: when the values of relevant features are highlighted further by increased variance, perhaps the intentional, social (as opposed to unintentional or nonsocial) presentation of options varied on these features will be further highlighted. Thus, we will not only investigate whether participants differ across conditions within Experiment 2 but also compare the behaviors within conditions between Experiments 1 and 2 to investigate whether the differences in feature variance (price) affected decision-making.

The second key difference comes after participants make their choice. In Experiment 2, we also asked participants to rate how important it was for them to have online access and print access to the newspaper on a scale from 0 (not important) to 100 (extremely important). The introduction of this additional measure in Experiment 2 was included to help further clarify whether or not the assumptions of Intentional Selection draw participants' attention to specific features of relevance (e.g., whether each access type is included), and therefore affect the perceived utility or importance of each access type. In respect to this, we would predict that within the Intentional condition, participants would rate print access as more important due to its purposeful inclusion among the options; as compared to the Accidental condition, where the presented did not intend to mark it as available, or compared to the Control condition, where sole access to a print-only option is absent and may suggest to participants that it is thus less relevant. Importantly, we also expect that these ratings would be similar between Accidental and Control conditions due to this shared inference between them; that print access is ambiguous and potentially not relevant.

#### Results

**Figure 3:**
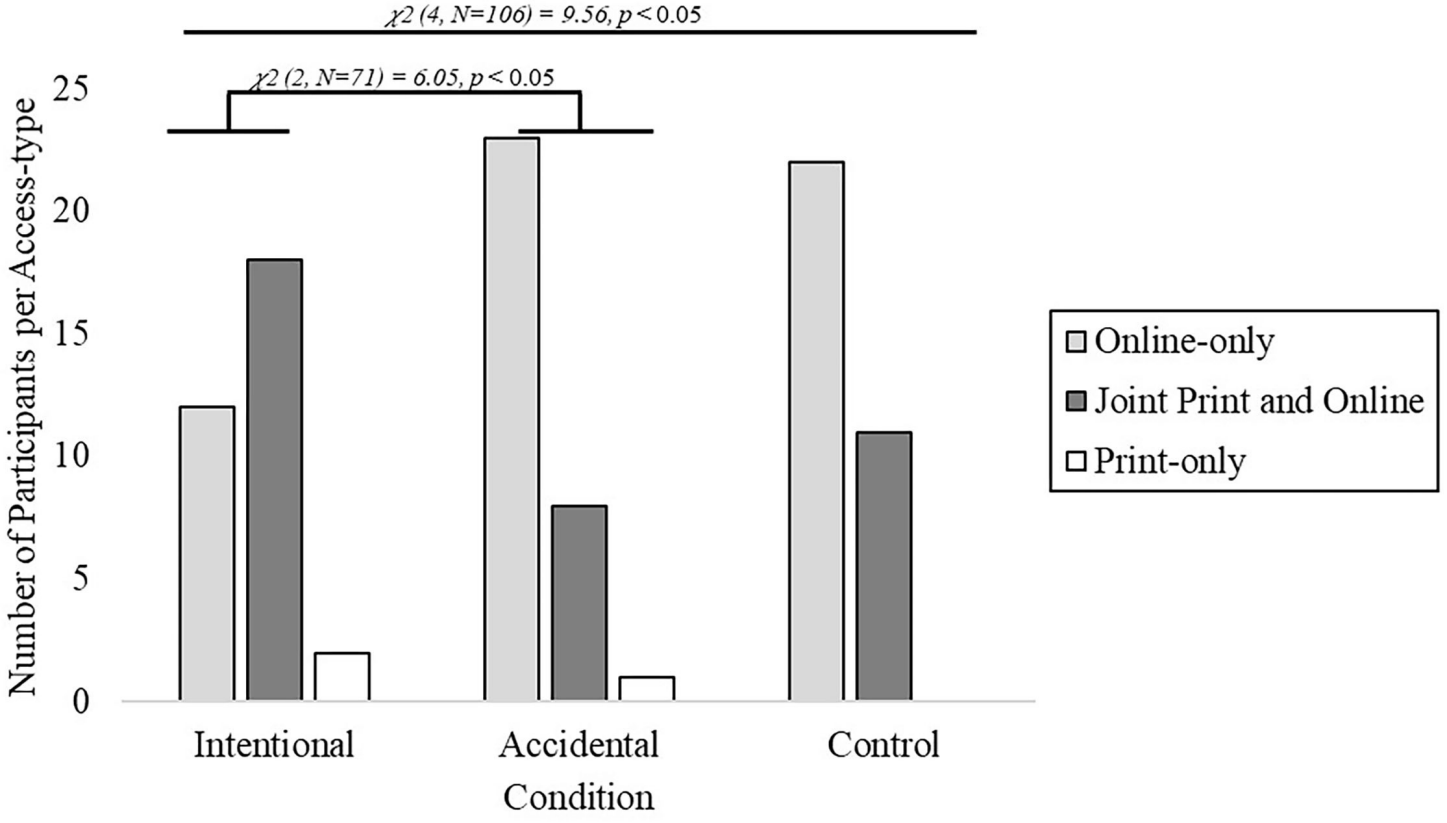
Forced choice results obtained from Experiment 2. Participants were more likely to choose the print and online subscription option and less likely to choose the online subscription option in the Intentional condition compared to Accidental and Control conditions, χ^2^ (4, *n* = 97) = 10.20, *p* = 0.037.

The number of participants who chose each plan differed across conditions [the chi-squared test of proportion; χ^2^ (4, *n* = 97) = 10.20, *p* = 0.037] replicating the results of Experiment 1. Specifically, participants in the Intentional condition were significantly more likely than the other conditions to choose the joint online and print subscription as well as less likely to choose the online-only subscription (see Figure 3). As in Experiment 1, Intentional and Accidental conditions were significantly different from one another despite having identical options available [the chi-squared test of proportion; χ^2^(2, *n* = 64) = 7.64, *p* = 0.022].

When further comparing the choices that are made of the two shared options (online-only and joint online and print subscriptions) across conditions, three additional chi-squared tests were performed. Here, we find that the Intentional condition has a significant difference from the Accidental condition [the chi-squared test of proportion; χ^2^(1, *n* = 61) = 7.29, *p* = 0.007], that the Intentional condition has a marginally significant difference from the Control condition [the chi-squared test of proportion; χ^2^(1, *n* = 63) = 3.49, *p* = 0.062], and the results find no significant difference between the Accidental condition and the Control condition [the chi-squared test of proportion; χ^2^(1, *n* = 64) = 0.15, *p* = 0.699]. Furthermore, after applying a Bonferroni correction for each of these pairwise comparisons (approximate α = 0.05/3 tests = 0.167), only Intentional and Accidental conditions remain statistically significant as noted (*p* = 0.007).

As in Experiment 1, we compared a preference between Intentional and Accidental conditions by examining the ratio of choices between online-only and the joint online and print subscriptions within the Control condition. Conducting the three comparisons using Fisher's Exact test, we find a significant difference between Intentional (choice ratio 12:18) and Accidental (choice ratio 23:8) conditions (one-tailed, *p* = 0.007). Importantly, the Intentional condition was found to be significantly different (one-tailed, *p* = 0.03) from the Control condition (choice ratio 22:11), but the Accidental condition (one-tailed, *p* = 0.35) was not significantly different from the Control condition.

Additional analyses were also performed to compare choice behavior between Experiments 1 and 2. Specifically, we looked at comparisons between analogous conditions—aiming to further analyze whether the Intentional Selection Assumption may be moderated by feature variance. Comparing within the condition between the two experiments, we find no difference in choice behavior between experiments across their Accidental [the chi-squared test of proportion; χ^2^(2, *n* = 67) = 1.88, *p* = 0.39] and Control [the chi-squared test of proportion; χ^2^(1, *n* = 68) = 0.94, *p* = 0.33] conditions. However, we do find a significant difference only between the Intentional Selection conditions of Experiments 1 and 2, χ^2^ [the chi-squared test of proportion; 2, *n* = 68 = 6.99, *p* = 0.03], suggesting that variability among features (e.g., price) may further modulate the sampling assumptions of Intentional Selection.

**Figure 4:**
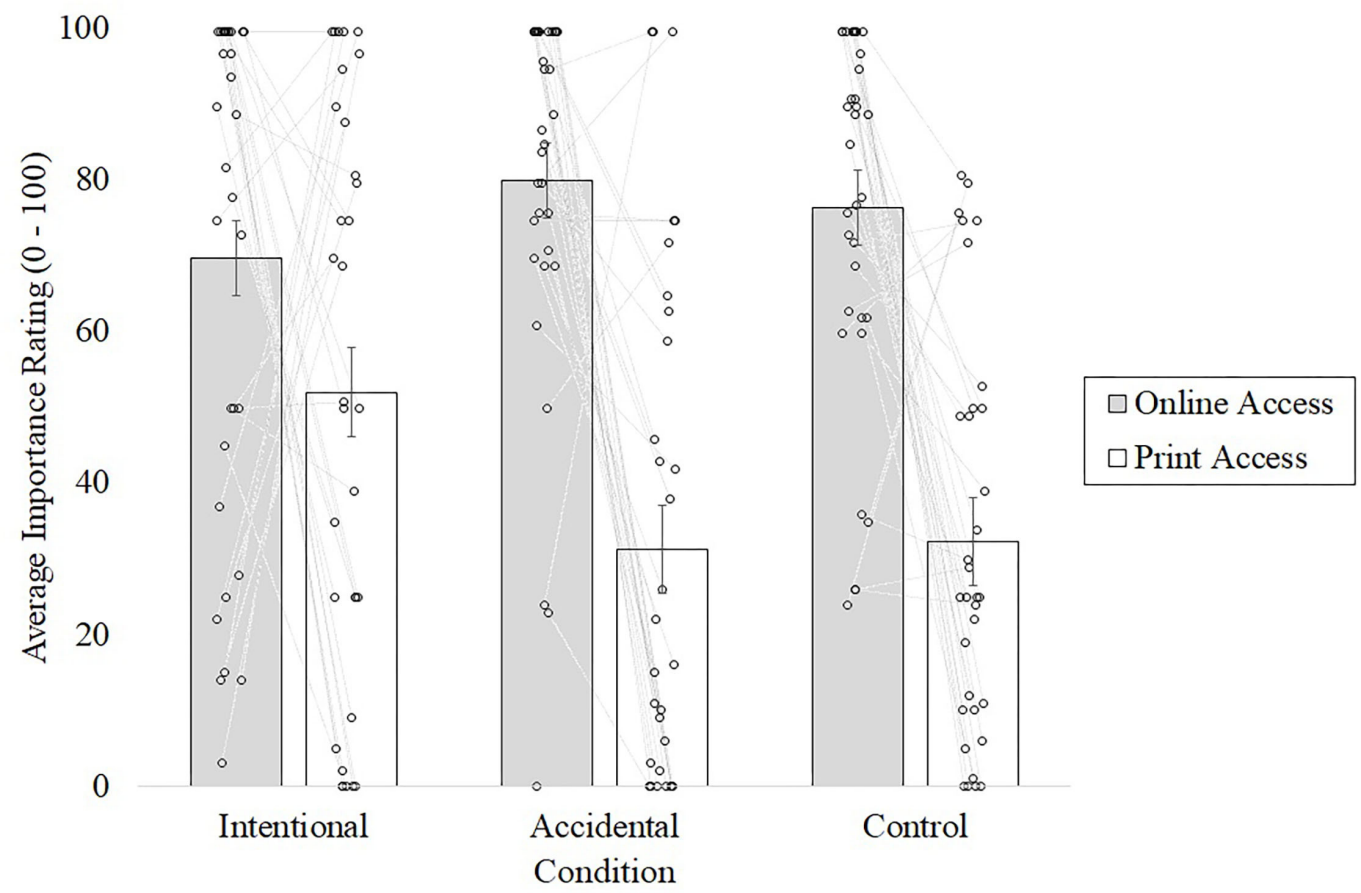
Participants' ratings of feature importance from Experiment 2. Error bars represent the SE per choice, within the condition. Each individual dot represents an individual participant's rating as per the respective column, with links between their ratings of the two access types—online and print. Here, participants rated the availability of “online access” similarly across conditions, *F*_(2, 94)_ = 1.09, *p* = 0.341, and η^2^ = 0.023. However, participants rated the availability of “print access” significantly higher in the Intentional condition, *F*_(2, 94)_ = 3.99, *p* = 0.022, and η^2^ = 0.078.

By comparing the importance ratings of having different types of newspaper access (Figure 4), there was no difference across conditions in how important online access was to them [one-way ANOVA; *F*_(2, 94)_ = 1.09, *p* = 0.341, η^2^ = 0.023]. Furthermore, there were significant differences in how important print access was across conditions [one-way ANOVA; *F*_(2, 94)_ = 3.99, *p* = 0.022, η^2^ = 0.078]. However, a planned contrast revealed that participants in the Intentional condition rated print access as more important than Accidental and Control conditions [*t*_(94)_ = 2.82, *p* < 0.01, *d* = 0.60].

Further investigation within the condition between the two importance ratings (online- and print-access) finds that participants in the Intentional condition only find online access (*M* = 69.6) marginally more important than print access (*M* = 51.9) [paired samples *t*-test; *t*_(31)_ = 1.58, *p* = 0.06, *d* = 0.49]. However, in comparison, both Accidental [paired samples *t*-test; *t*_(31)_ = 6.04, *p* < 0.0001, *d* = 1.61] and Control [paired samples *t*-test; *t*_(31)_ = 5.92, *p* < 0.0001, *d* = 1.73] conditions claim that they find online access (*M* = 79.8 and 76.2, respectively) significantly more important than print access *(M* = 31.2 and 32.2, respectively). These results regarding participants' ratings of importance (across conditions) are consistent with our hypothesis of the Intentional Selection Assumption. Specifically, because participants in Accidental and Control conditions were not intentionally given information about the value of print access (either through a misrepresentation or through an omission), they would not have the same reason as those in the Intentional condition to infer that print access is something valuable to possess.

Finally, additional linear regressions were performed to investigate whether the higher participant importance ratings of the print access that was predicted whether they chose an option with print access (the feature affected by the manipulation). Here, participants' importance ratings for print remained as they were collected (from 0 to 100) as the predictor variable, and the dependent variable was coded as either 0 (when choosing the online-only subscription option) or 1 (when choosing either options that included the print access). The dependent variable includes both the options with print access due to a low frequency of participants choosing the print-only access option across conditions. We find that, across all three conditions, the importance of print access predicts the decisions to purchase an option with print access for all participants [*F*_(1, 95)_ = 181.75, *p* < 0.001, *R*^2^ = 0.653] and within condition [Intentional condition, *F*_(1, 30)_ = 77.73, *p* < 0.001, *R*^2^ = 0.722; Accidental condition, *F*_(1, 30)_ = 54.47, *p* < 0.001, *R*^2^ = 0.645; Control condition, *F*_(1, 31)_ = 35.71, *p* < 0.001, *R*^2^ = 0.535].

Following this, in comparing the strength of the relationship between participants' importance ratings and final choices (*via* Fisher's *R*-to-*Z* transformation), we find no significant difference regarding this relationship between Intentional (Pearson's *R* = 0.803, *p* > 0.05) and Accidental (Pearson's *R* = 0.803, *p* > 0.05) conditions (*z* = 0.55, *p* = 0.29). Furthermore, we find no significant difference between Control and Accidental conditions (*z* = −0.67, *p* = 0.25) but find a marginally significant relationship difference between Intentional and Control conditions (*z* = 1.23, *p* = 0.10). Thus, while the results between conditions here are only marginal, these findings are consistent with the previously ran planned contrast, finding that participants in the Intentional condition rated print access as more important than the contrasting conditions—importantly, when compared the Accidental condition.

Together, these findings from both Experiments 1 and 2 suggest that the Intentional Selection Assumption may have affected participants' valuation of option features, and thus their choices in the Intentional condition. By comparing these results to those of Experiment 1, it also suggests that increasing the price of an online subscription option decreased the relevance of the price feature of that option and in turn increased the difference between Intentional and Accidental conditions. Because the difference in the price feature of each option was now lower, participants in the Intentional condition may have attended more to the generation of their set of available options and thus inferred an increased utility of print access.

## Discussion

Our goal was to further highlight the importance of social cues in decision-making as found in past research. However, *via* our proposed Intentional Selection Assumption, we looked to address the importance of sampling assumptions that individuals may be inferring when deciding—despite past experimental designs' implicit assumption of random sampling.

Participants may have a fixed notion of feature weight; however, the relevance of those features may be context-dependent. Specifically, the options presented to participants may have been intentionally selected by a social agent with a specific goal in mind, providing the participants with additional information about the feature relevance that may not be available in other choice contexts. Therefore, these participants can make inferences about the relevance of features and inferences about the goals of the presenter, altering their choice behavior. However, if participants are presented with options without intention or in a non-social context (e.g., randomly), these same inferences may not be expected of them.

Our account of intentional sampling was followed by the evaluation of our proposal among a simplified demonstration (Appendix) and two decision-making studies. These experiments tested whether the social context within each decision-making scenario affected the participant's final choices, highlighting how the Intentional Selection Assumption explains differences in the chooser's inferences about another person's beliefs. In our experiments, participants' decision-making behavior was tested. As in classical decision-making tasks, participants had to intuitively calculate and weigh the normative utility of various options within a presented set, differing in price and content. All participants had been given similar options across conditions, and only received differing information regarding the social context of the presentation of all options; in particular, whether the options were provided intentionally or accidentally. When all options had been intentionally presented, participants' choice behavior reflected this, by showing through their higher appraisal for said option. However, when some of the options were presented accidentally, participants' appraisal and preference for the options affected by this change in presentation also changed. Because the questioner within the intentional condition intentionally presented the options to participants, they may have inferred the importance of the presence, price, and media format of all features. However, participants in the Accidental condition may not have made the same inferences about the features; because the print-only option was not intentionally presented, it should not be similarly valued as it is in the intentional condition. Thus, participants in the Intentional condition where all information were intentionally presented may have performed different calculations from the contrasting conditions; highlighted not only by the final decisions in Experiments 1 and 2, but further so by their ratings of feature importance (media-type access) in Experiment 2.

### Comparison to Contemporary Models and Effects of Context-Dependent Choice

An investigation of framing effects has been incredibly fruitful in researching decision-making over the past century. Importantly, strides have been made in investigating the nuances of framing effects and contextual choice as affected by the many variable parts of creating a decision-making task (Johnson et al., 2012). However, older models of choice that may have centered their principles within contextual decision-making on a variation in outcome certainty or branching may not entirely account for the differences we find in our experiments. In particular, we may see that the differences between Intentional and Accidental conditions would not be predicted to occur as they did in our experiments according to past models and theories of contextual choice.

For example, Tversky and Kahneman's (1992) Cumulative Prospect Theory (CPT) has been pivotal in the development of context-dependent decision-making theories and models as it highlights systematically occurring “anomalies” of choice as a result of such variance. Within our tasks, participants do experience a major phenomenon as described by CPT that violates standard models (at the time)—a framing effect. However, CPT may have predicted in our experiments that participants would be indifferent between the Intentional and Accidental conditions, given that both scenarios presented the same options at the same explicit values. There are technically no differences in prospects between these two experimental conditions yet the differences in choice behavior (Experiments 1 and 2) and even explicitly the ratings of feature importance (Experiment 2) occur. Similarly, the transfer of attentional exchange model (TAX; Birnbaum and Navarrete, 1998; Birnbaum, 2008a,b) should have similar expectations of outcomes as CPT in our scenarios, albeit for different reasons due to its differing valuation mechanism. Specifically, in our scenarios, there are no gambles or probabilistic variances that would allow for branching to occur differently between conditions. Thus, as in CPT, the TAX model may also predict similar decision-making across conditions. However, once more, we instead found differences between conditions despite the lack of branching. Another foundational model of choice, Decision Field Theory (DFT; Busemeyer and Townsend, 1993), may also face the same challenges like CPT and TAX. Therefore, we suggest that perhaps the addition of the social cue of Intentional Selection and presentation (as opposed to accidental or unintentional) may have played a role in the differences between conditions that are not fully captured by models such as CPT, TAX, or DPT.

Furthermore, one might be concerned that, by adding this accidental manipulation to the context, we confound our experiment with an alternative explanation—namely that accidental events may also be more scarce than intentional ones. Specifically, the Scarcity Effect predicts that the selectors will be more likely to choose an item perceived as harder to come by Cialdini (1993) and Worchel et al. (1975). However, in this study, the predictions based on the Scarcity Effect were only weakly (if at all) seen between both the experiments. For example, <10% of the participants in the Accidental condition in Experiment 1 chose the print-only subscription option. This option may have been encouraged due to a pragmatic cue that the option was scarce and therefore valuable, with said value or rarity highlighted by its “accidental” inclusion. Furthermore, as seen in the ratings of importance for each type of access from Experiment 2, it was actually the Intentional condition (the rating of approximately 52/100) that rated the print-only option as important; compared to the conditions where said option may have been “potentially scarce” (Accidental condition rating of 31, Control condition rating of 32). Thus, future work may instead be tasked with disentangling how rarity and importance differently affect the perceived value.

In returning to contemporary informational accounts, we can analyze our results based on the predictions they may make. For example, when considering past accounts on the Decoy Effect (e.g., Wernerfelt, 1995; Kamenica, 2008), one may argue that the salience of the print-only option may have been heightened in the Accidental condition by being “highlighted” through a mention of its accidental inclusion. Thus, by a prior work on the Decoy Effect and an earlier discussion of salience effects (e.g., Bordalo et al., 2013), if all else follows, we might expect that participants within the Accidental condition would thus apply a “disproportionately high weight” to the salient attribute (the highlighted availability of print access) and both choose the options with print access more often, while we also explicitly rate the importance of print as higher than in contrasting conditions where it was less salient (e.g., plainly presented as available in the Intentional condition). However, the results obtained from both experiments revealed that this canonical description of the Decoy Effect may not always be the case—as the Accidental condition actually sought out print access less often (by choosing either the joint print and online or the print-only option) than the Intentional condition despite the feature values being equivalent, potentially resulting in a novel version of the “Decoy Effect” that may instead depreciate the value of the highlighted feature. Furthermore, the Accidental condition (in particular for Experiment 2) was notably similar in behavior to the condition where print access as a feature would be less salient, the Control condition, due to the exclusion of print-only subscriptions as an available option. Thus, we see that, despite the heightened salience of a feature or an option within the Accidental condition *via* the design of our experiments (highlighting the accidental inclusion of the print-only subscription option), the difference in social cues (intentional vs. unintentional inclusion) elicited the opposite of what would be predicted by the work theory within the Decoy Effect; similar to the outcomes as predicted prior in the discussion of the Scarcity Effect.

Further insights into the differences between the Intentional Selection Assumption and the Decoy Effect can be drawn from the additional analyses in Experiment 2. Despite the inclusion of a “strictly dominated” decoy option (print-only access) in Intentional and Accidental conditions, participants in the Intentional condition rated the importance of the highlighted feature higher than in the Accidental condition. Furthermore, while the results for our regression analyses were only marginal between conditions, the rated value of print access was still higher for the Intentional condition compared to the Accidental and Control conditions. Future work could investigate how social-contextual cues (including but not limited to the Intentional Selection Assumption) affect valuation as predicted by normative values. Power analysis from our studies suggests that future work should seek out larger samples when performing future projects such as those described recently.

Following this, it is also important to discuss salience-driven models (e.g., Spitmaan et al., 2019) that have highlighted the importance of attention in further describing human decision-making. Here, salience and attention mechanisms are proposed to differentially weight options and their values. However, the calculated values and the decisions that are made may be made in terms of gains and losses (e.g., prospects per CPT; Tversky and Kahneman, 1992). In respect to the Intentional Selection Assumption, we may find congruence between the scenarios presented by Spitmaan et al. (2019) and our experimental results. For example, as mentioned earlier in regard to the potentially higher salience of the “print-only” option within the Accidental condition, the difference in salience between this and the Intentional condition may be modulated similarly to the decisions in the gambles as described by Spitmaan et al. (2019). Thus, investigation into whether salience and social cues mingle, affecting decision makers' inferences, may be a fruitful endeavor for future experiments and formal modeling.

### Future Work and Limitations

Further future work may also consider how social inferences as affected by the Intentional Selection Assumption may alter choice behavior involving other classical decision-making phenomena. For example, the Endowment effect (Kahneman et al., 1991) assumes that most individuals have stable, well-defined preferences and make choices consistent with those preferences. When an individual is given an endowment, they are typically reluctant to trade it for another item of similar value or sell it for its described market price. In some cases, an individual may receive an endowment and outright refuse to trade it away, regardless of what they may be offered in return. This Endowment effect can be found even when the object endowed is trivial and easily attained. For example, in an experiment conducted by Kahneman et al. (1991), participants were unwilling to sell both coffee mugs and pens for <2 times their relative market prices (as described by the experimenters). This Endowment effect may be possible to explain through the Intentional Selection Assumption. If a presenter can successfully “endow” a decision maker with a gift that matches the endowed individual's preferences or is claimed to be highly individualized for them (e.g., through a personality test), the Endowment effect may hold, and individuals may refuse to trade away this endowment for an alternative. However, if the endowment is gifted without any social implications (e.g., randomly assigned), the effect may not occur, and the endowed individual may be willing to part with their endowment.

One can also imagine the benefits of understanding option presentation in machine learning. Similar issues regarding how information is presented are prevalent in its applications, such as within the framework of recommender systems (Yang et al., 2017). We can consider the importance of both context and presented options when considering online marketplaces and their algorithms' reliance on the consumer to provide examples of products they are both interested in and would actually buy. This problem can be seen as similar to those presented to young learners in active learning paradigms. Here, the problem faced in regard to learning the concept of what a customer would want.

However, an issue may arise when remembering that our proposed Intentional Selection Assumption requires a chooser to infer the beliefs and goals of another person. Specifically, due to this social inference, there may be a discrepancy between the actual goal of the presenter and what is being inferred as the goal by a chooser (Jones and Nisbett, 1972). For example, we can consider a job recruiter looking at resumes and applications for their company's recent advertisement. These submitted resumes can vary in length and content. Some may be highly detailed with a long list of every accomplishment, both big and small. Some may be shorter and more concise, listing only the very best achievements. Research has found that individuals in a position to rate and review such resumes preferred the shorter list, proposing that they intuitively calculate an average across all items on the resumes instead of considering the “sum” of the listed accomplishments (Weaver et al., 2012). Therefore, some of the lower-valued, positive accomplishments actually distract and hinder the higher-valued, positive ones.

This is known as the Presenter's Paradox, an example of potentially disconnected views between a presenter and an evaluator regarding what may be considered important in any presentation. Presenters seem to find that the properties of an object are additive and that positive values should only help increase the overall perceived value. But, this work has found that the evaluators perceive the presentations as averaging, hindered by the inclusion of the lesser-known attributes. These effects are also found when making judgments about product purchasing, negative punishments, and curriculum vitae (Weaver et al., 2012; Powdthavee et al., 2018). Thus, we may benefit from identifying the differences in the inferences made between the presenter and the appraiser (decision maker), in respect to these examples here, to achieve the goal of adequately describing theories and models of choice.

Finally, some may argue that we found a potentially modulating effect of feature variance (the price value of options) between Experiments 1 and 2^2^. For example, the price of the online-only option was altered between our experiments—from $59 to $99. This difference between experiments led to a stronger effect in Experiment 2, suggesting that perhaps feature variance across scenarios may have a modulating effect that interacts with the framing effects elicited by the Intentional Selection Assumption. Thus, future research may consider investigating the different ranges of feature variance (e.g., the same scenario but varied with a range of prices instead of the two from our experiments) to investigate potential interactions with the Intentional Selection Assumption. Additionally, a deeper insight into how the contextual effects of the Intentional Selection Assumptions potentially affect normative values, such as participants' perceived values for each of the access types (print and online), can be further investigated by modifying the ranges of the rating procedure. For example, instead of a strictly positive range of Experiment 2's rating paradigm (from 0 to 100), future work may extend into the negative range (e.g., −100 to 100) to investigate whether there is an effect when scenarios may be averse to either access type (e.g., an individual may be averse to online access if attempting to reduce screen time; another person averse to print access if they are attempting to be eco-friendly and reduce paper waste).

## Conclusion

As described earlier, there exist many human behavior anomalies that deviate from what theories may believe to be “optimal” or “rational.” Within the decision-making literature itself, it is famously noted that an empirical result qualifies as an anomaly if it is difficult to rationalize or if implausible assumptions are necessary to explain it within a paradigm (Kahneman et al., 1991). Therefore, some anomalies, as described earlier, may have arisen from intentional manipulations to the framing of the decision-making scenario (e.g., modified Ultimatum Games; FeldmanHall et al., 2014), or the features of the options that are decided upon (e.g., equivalent information appraisals; Leong et al., 2017). However, here, we discuss and test the plausibility of whether intentionality is being inferred by participants when past experiments had not intended so in their designs. Thus, we propose that the Intentional Selection Assumption is a key aspect of decision-making that may be important to account for future accounts when working toward a comprehensive understanding of decision-making.

## Data Availability Statement

The data sets generated and analyzed for this study can be found in the Open Science Framework Storage (USA) [https://osf.io/xzjg8/?view_only=bdbe7ae1238e46f89a5e4f4b7c3e78cf].

## Ethics Statement

The studies involving human participants were reviewed and approved by Rutgers University Institutional Review Board. The patients/participants provided their written informed consent to participate in this study.

## Author Contributions

JC, KD, LC, PS, and EB: conception or design of the work, data analysis, interpretation, and final approval of the version to be published. JC, KD, and LC: data collection. JC and EB: drafting the article and critical revision of the article. All authors contributed to the article and approved the submitted version.

## Funding

This research was supported by funding from the NIH award 5R25GM096161 supporting JC, National Science Foundation, CAREER Grant DRL-1149116 to PS, the Choosing to Learn Grant SES-1627971 to EB, and the Science of Learning Grant SMA-1640816 to EB and PS.

## Conflict of Interest

The authors declare that the research was conducted in the absence of any commercial or financial relationships that could be construed as a potential conflict of interest.

## Publisher's Note

All claims expressed in this article are solely those of the authors and do not necessarily represent those of their affiliated organizations, or those of the publisher, the editors and the reviewers. Any product that may be evaluated in this article, or claim that may be made by its manufacturer, is not guaranteed or endorsed by the publisher.

## Footnotes

^1^In this proof-of-concept experiment, we manipulate whether participants see items as presented to them intentionally or randomly, then ask participants to infer what the next selected option would be. Here, participants show significant differences between scenarios (Intentional and Random), demonstrating they may be sensitive to the difference between intentionally and randomly drawn options.

^2^We thank the anonymous reviewers for suggesting these future extensions of the experiments.
