# Supplementary material for: The Intentional Selection Assumption
Source: Front Psychol. 2021 Oct 26;12:569275. doi: 10.3389/fpsyg.2021.569275 (PMC8576492; doi:10.3389/fpsyg.2021.569275)
Supplement: Supplementary file 1 [file Presentation_1.zip › Appendix_ColantonioDurkinEtAl.pdf]

1 Appendix: Empirical demonstration of intentional inference

1.1 Experiment

This preliminary experiment was designed as a simple test of our initial intuitions about the importance of feature relevance within different choice contexts. The goal is to provide a proof of intuition that an individual's expectations about missing samples in a set following intuitions about how intentionally and accidentally sampled sets differ. Specifically, when trying to determine relevant features, a set of options that is intentionally sampled would contrast among those features. For example, if the feature red is relevant, a questioner with the intention of highlighting this fact may provide a set of options that includes options that are red and options that are not red. However, a non-social agent may not ensure that such variability is conveyed in the options presented to the chooser. For example, they may pick options randomly.

To test this basic intuition of intentional sampling, we gave participants a task in which information about one of the sampled options was missing and provided participants a simple forced choice response to fill in the missing information. Our design contrasted a case in which the options were known to be intentionally sampled for the participant to a case in which the options were known to be randomly sampled. Critically, the dependent variable (which missing feature went with the obscured final option) is predicted to strongly favor one feature in the intentional condition only. If participants are sensitive to these differences in sampling, we predict differing patterns of responding for the conditions.

1.1.1 Participants

95 workers were recruited from Amazon Mechanical Turk. An initial payment of $0.10 was offered for completion of the two-minute study but was raised to $0.50 to encourage recruitment. Twelve participants failed to pass an attention check and were therefore excluded from any analysis. The 83 remaining participants were randomly assigned to one of two experimental conditions: the Accidental condition (n = 43) and the Intentional condition (n = 40).

1.1.2 Procedure

Participants were required to provide a forced choice response regarding a novel object, labeled a "Widget". Specifically, participants read the prompt: "Widgets are objects that can vary on shape (Triangle or Square) and color (Blue or Red)." However, the text following this prompt differed between the two conditions. In the Accidental condition, participants were told: "A coin is flipped to decide shape, and then another coin is flipped to decide color, so the process is random. The Widget factory is making Widgets by first flipping the shape coin and then flipping the color coin." However, in the Intentional condition, participants are told: "Your friend, Alex, is at the Widget factory and can bring one home for you. Alex wants to know what kind of widget you would prefer." In both conditions, participants are shown the prompts "Widget 1 is Triangular and Blue," "Widget 2 is Square and Red," and "Widget 3 is Triangular and [TEXT CUT OFF]." We then collected our main measure, having participants complete a forced choice for the question, "Do you think Widget 3 is blue or red?"

1.1.3 Results and Discussion of Preliminary Experiment

Our analysis began by comparing whether participants differed in choosing red or blue as the color for Widget 3, between conditions (Figure 5). Using a chi-squared analysis, we found significance between conditions, $\chi^2(1, N = 83) = 11.62, p < .001$.

As we predicted, in the Intentional condition, participants chose "red" as Widget 3's color significantly more than predicted by chance (N selecting red = 28 of 40; binomial, $p < .01$). This result indicates that participants expected items to be meaningfully selected such that options were not redundant. Such an inference demonstrates sensitivity to intentional sampling.

In contrast, in the Accidental condition, participants chose "blue" significantly more than predicted by chance (N selecting red = 14 of 43; binomial, $p < .05$). Although we did not have strong a priori predictions that participants would choose differently from chance in this condition, the bias to choose "blue" in the Accidental condition is consistent with previous research finding that adults over-attribute alternation to random events (Reichenbach, 1934; Bar-Hillel & Wagenaar, 1991).

Critically, the significant differences between conditions provides a simple demonstration of human sensitivity to Intentional versus Accidental conditions. This serves as a test of the first criteria in establishing whether these intuitions support choice preference among intentionally versus accidentally sampled items explored in Experiments 1 and 2. Participants were informed of different items produced at a factory, either by a social agent with a goal (their friend buying a gift) or a non-social agent following an algorithm (a computer flipping a coin). When faced with a forced choice question (choosing red or blue), normative accounts would posit that both choices had equal likelihood of occurring (50%).

Within our design, there may be concern regarding participants' bias towards specific colors, as the two sampled Widgets were consistent: all participants are always shown the Blue Triangle and Red Square before choosing the final Widget color. For example, some recent work finds that participants are capable of learning about relationships between covariant features (e.g., color and object orientation; Thakur et al., 2021) both explicitly and implicitly, and can affect subsequent decisions made based on these previously learned associations. Thus, we might have predicted that participants in both conditions would similarly have used the sampled object colors as cues for the "correct" answer and chosen the same color. However, our results find that the framing of the two conditions' sampling methods (intentionally by a friend versus randomly by a machine) does not exhibit the same choice bias between the two conditions.

In fact, when contrasting conditions, it seems as though behavior within the Accidental condition exhibits sensitivity to biases - whether it be the over-attribute alternation to random events (e.g., Bar-Hillel & Wagenaar, 1991) or associated features (e.g., with color; Thakur et al., 2021). Thus, the inclusion of a social cue may have significantly changed the sampling inferences made by the participants, as they may have inferred the goal of only providing informative samples within the intentional social condition, but not in the accidental, non-social condition. This difference between conditions revealed an initial, simple test of the effects of the sampling processes on participants' choice.

2 Figures

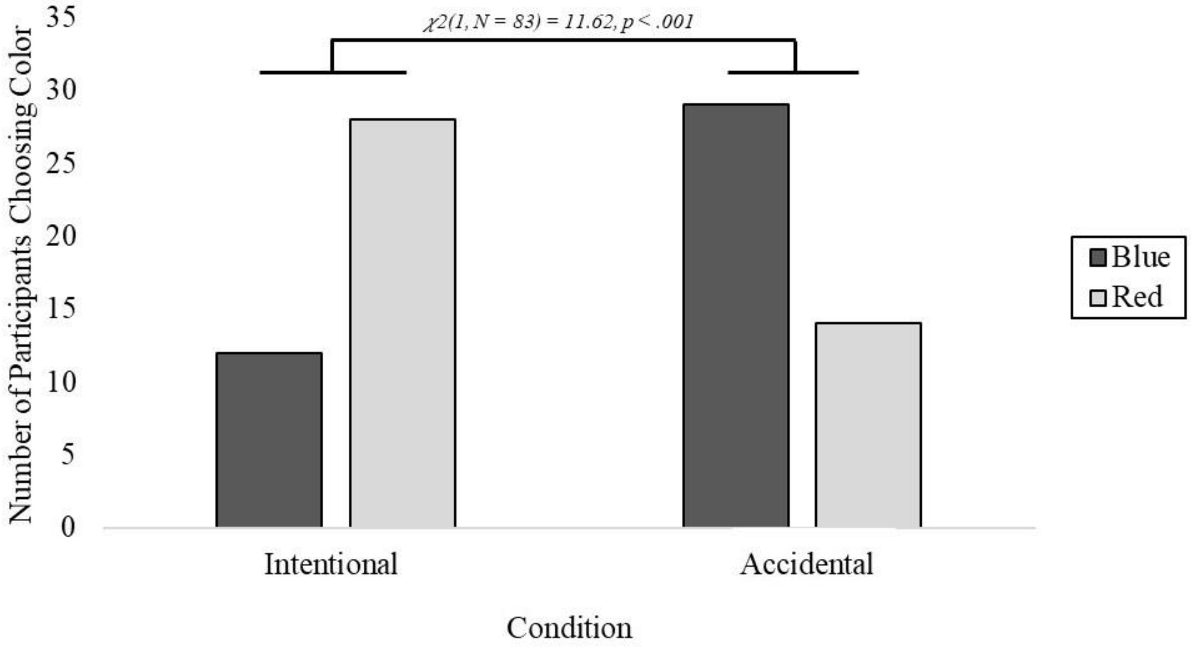

Figure 5. Results from preliminary experiment. Participants in the Intentional condition were more likely to choose "red" as the next Widget, while participants in the Accidental condition were more likely to choose "blue", $\chi^2(1, N = 83) = 11.62$, $p < .001$. Furthermore, when comparing both conditions against chance (p(Picking Red) = 0.50), we find that the Intentional condition picked "Red" significantly more often than predicted by chance (N selecting red = 14 of 43; binomial, $p < .01$) and that the Accidental condition picked "Red" significantly less often than predicted by chance (N selecting red = 14 of 43; binomial, $p < .05$).
