# Supplementary figures and images for: The Intentional Selection Assumption
Source: Front Psychol. 2021 Oct 26;12:569275. doi: 10.3389/fpsyg.2021.569275 (PMC8576492; doi:10.3389/fpsyg.2021.569275)

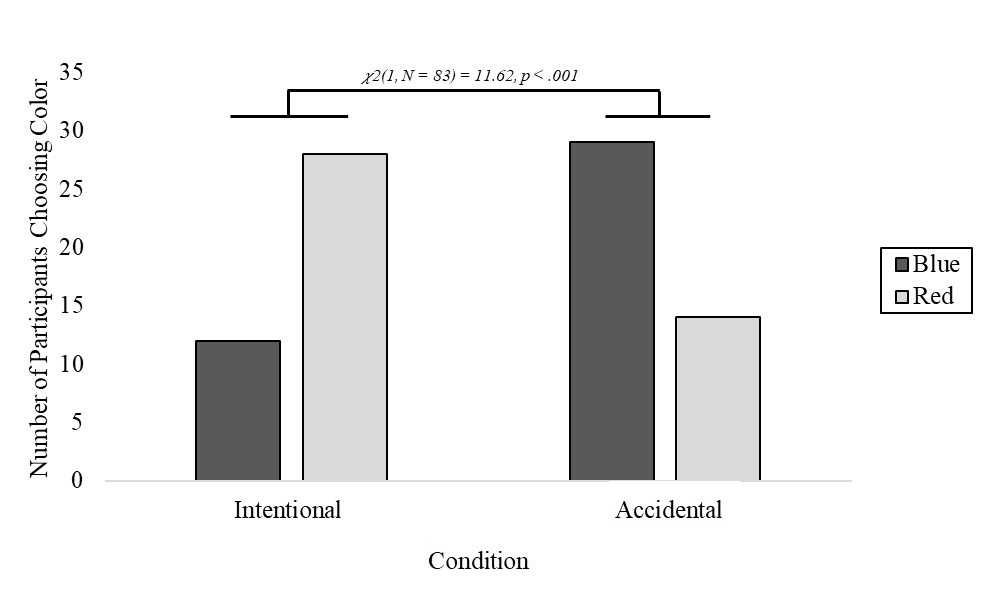

Supplement: Supplementary file 1 [file Presentation_1.zip › Figure5.jpg]
